# Synovitis, Acne, Pustulosis, Hyperostosis, and Osteitis Syndrome with Purely Osteolytic, Not Osteosclerotic, Lesions Mimicking a Malignant Tumor

**DOI:** 10.1155/2020/6316921

**Published:** 2020-03-28

**Authors:** Hideyuki Kinoshita, Takeshi Ishii, Hiroto Kamoda, Yoko Hagiwara, Toshinori Tsukanishi, Sumihisa Orita, Kazuhide Inage, Naoya Hirosawa, Seiji Ohtori, Tsukasa Yonemoto

**Affiliations:** ^1^Department of Orthopedic Surgery, Chiba Cancer Center, 666-2 Nitonacho, Chuo-ku, Chiba 260-8717, Japan; ^2^Department of Orthopaedic Surgery, Graduate School of Medicine, Chiba University, 1-8-1 Inohana, Chuo-ku, Chiba 260-8670, Japan

## Abstract

Synovitis, acne, pustulosis, hyperostosis, and osteitis (SAPHO) syndrome is a rare inflammatory disorder with multiple phenotypes. The syndrome has identifiable radiologic characteristics that are the most important when making a diagnosis. X-rays of cases diagnosed with SAPHO syndrome reveal sclerotic lesions or mixed lytic and sclerotic lesions. Pure osteolytic lesions in SAPHO syndrome are rare, and to the best of our knowledge, no study has reported the radiologic change of purely osteolytic lesions to osteosclerotic lesions over time. Herein, we report on the case of a woman experiencing severe left thigh acute pain and having a medical history of palmoplantar pustulosis. Although SAPHO syndrome was suspected because of palmoplantar pustulosis, based on radiologic findings, bone metastasis of a malignant tumor or chronic bacterial osteomyelitis owing to a purely osteolytic lesion was suspected. However, needle biopsy revealed no malignancy and bacterial culture was negative, thus suggesting SAPHO syndrome. Nonsteroidal anti-inflammatory drugs, bisphosphonates, and corticosteroids were administered, which improved the left thigh pain. Furthermore, the radiologic change of osteolytic lesions to osteosclerotic lesions over time was confirmed, leading to the diagnosis of SAPHO syndrome. Our case demonstrates that knowledge of atypical radiologic findings is necessary to diagnose initial SAPHO syndrome.

## 1. Introduction

Synovitis, acne, pustulosis, hyperostosis, and osteitis (SAPHO) syndrome was first described by a group of French rheumatologists in 1987 [1]. In addition, Chamot et al. reported “le syndrome acne pustulosehyperostoseosteite” and introduced the acronym SAPHO to designate these five frequently combined disorders. Many different names, including sternocostoclavicular hyperostosis, acne-associated spondyloarthropathy, and pustuloticarthro-osteitis, have been used for this syndrome [2]. Radiologic findings reveal that hyperostosis is highly characteristic of SAPHO syndrome and is included in the diagnostic criteria of SAPHO syndrome [3]. Sclerotic lesions or mixed osteosclerotic and osteolytic lesions of the bone are typical findings of many SAPHO syndrome cases. To our knowledge, pure osteolytic lesions of SAPHO syndrome are rare, with no reports on the radiologic change of purely osteolytic lesions to osteosclerotic lesions over time.

Here, we present a case of SAPHO syndrome with purely osteolytic, not osteosclerotic, lesions clinically suspected to be a malignant tumor. Because initial radiologic findings and diagnostic criteria of SAPHO syndrome were contradictory, the diagnosis and treatment of the case were difficult.

## 2. Case Presentation

**Figure 1:**
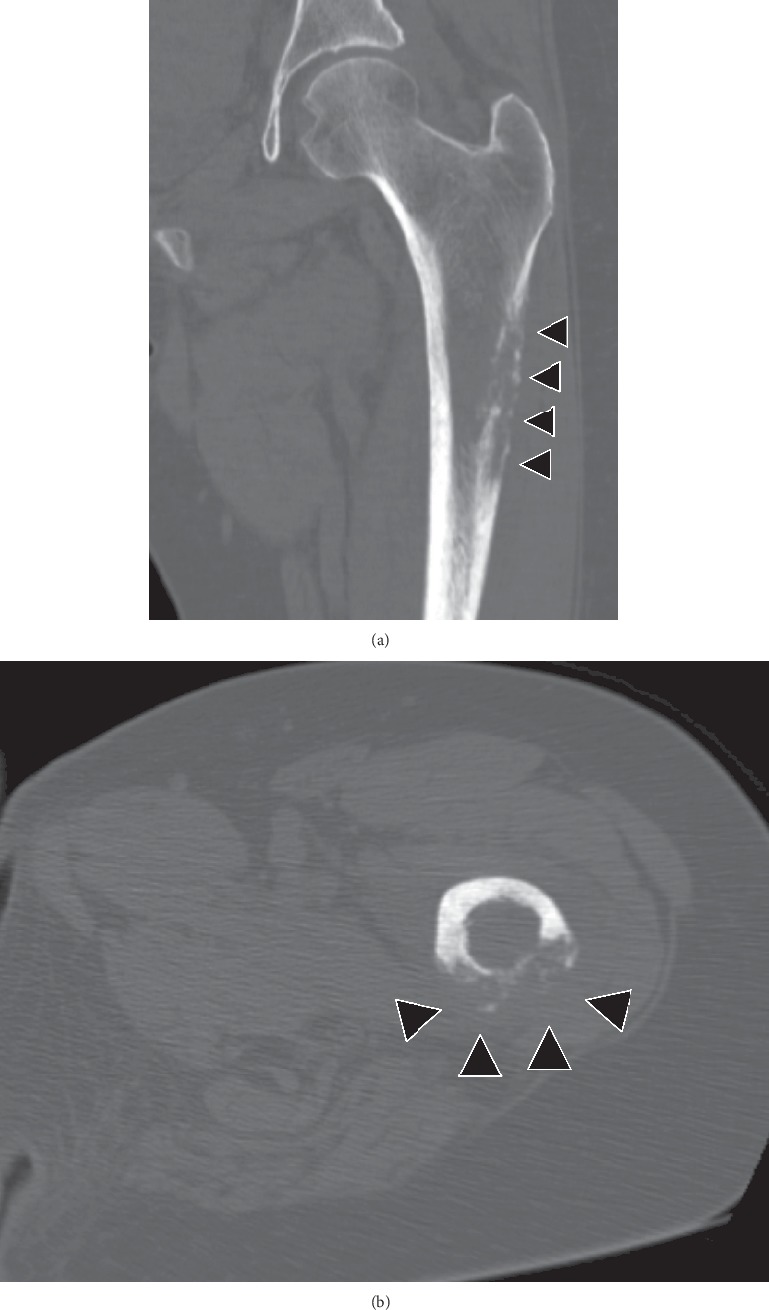
Computed tomography at first visit showing a purely osteolytic lesion on the left femur (arrowhead). (a) Coronal view and (b) axial view.

**Figure 2:**
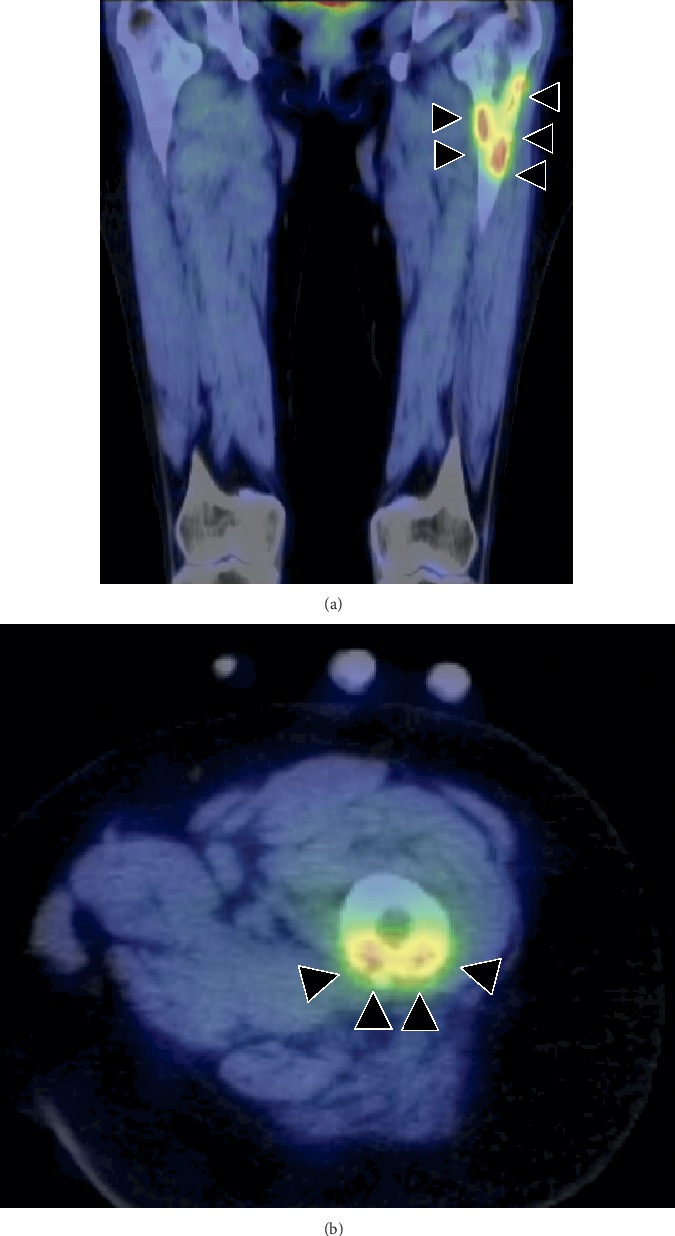
Whole-body PET/CT showing 18-FDG accumulations in the left femur only, suggesting a primary malignant bone tumor or chronic bacterial osteomyelitis (arrowhead). (a) Coronal view and (b) axial view.

**Figure 3:**
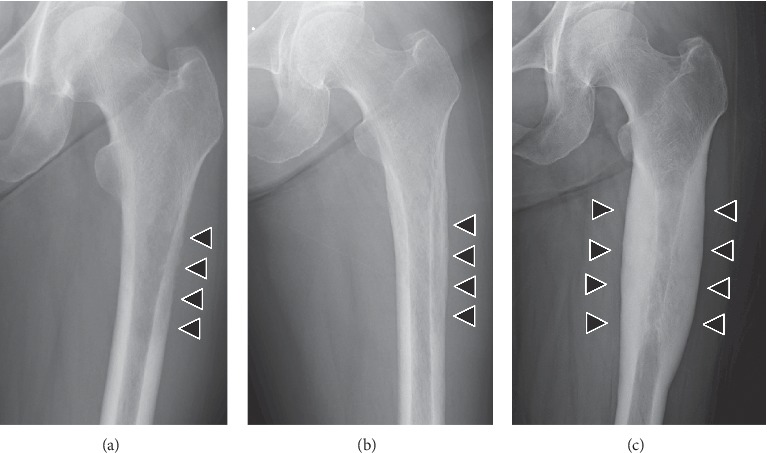
X-ray findings of the left femur at different timepoints showing the radiologic change of a purely osteolytic lesion to an osteosclerotic lesion over time (arrowhead). (a) At first visit, (b) at 3 months after the first visit, and (c) at 15 months after the first visit.

A 54-year-old woman with a 2-month history of left thigh pain presented to our orthopedic outpatient department. She had no medical history of preceding trauma except for palmoplantar pustulosis. On physical examination, were no signs of infection or rash on the left thigh, except for thigh pain. Computed tomography (CT) revealed a severe lytic lesion on the left femur, suggesting bone metastasis (Figure 1). Blood test results revealed mildly elevated serum alkaline phosphatase and C-reactive protein (CRP) concentrations at 473 (normal: 106–322) IU/L and 1.1 (normal: 0–0.14) mg/dL, respectively. Conversely, tumor markers were normal. Although SAPHO syndrome was suspected based on palmoplantar pustulosis, we suspected bone metastasis of a malignant tumor because radiologic findings did not reveal a sclerotic lesion or a mixed osteosclerotic and osteolytic lesion but revealed a purely osteolytic lesion. Whole-body positron emission tomography/CT (PET/CT) showed 18- fluorodeoxyglucose (FDG) accumulations only in the left femur, suggesting a primary malignant bone tumor or chronic bacterial osteomyelitis (Figure 2). CT-guided needle biopsy of the left femur was performed, and histopathological examination using hematoxylin and eosin (HE) staining revealed woven bone matrix in the cortex and fibrosis in the bone marrow cavity, some of which formed the necrotic bone, thus suggesting chronic osteomyelitis. Furthermore, HE staining of the specimen revealed no malignancy or tumor lesion, and the bacterial culture was also negative. Nonsteroidal anti-inflammatory drugs (NSAIDs) were administered for pain control, and oral bisphosphonates were introduced based on reports of their successful use for treating SAPHO syndrome [4]. X-ray in the 3 months after the first visit confirmed the presence of a sclerotic lesion of the femur (Figures 3(a) and3(b)). The pain gradually disappeared following treatment with corticosteroids (prednisone 5 mg⁄day). X-ray at 15 months since the first visit revealed a severe circumferential sclerotic lesion of the femur, which is typically found in SAPHO syndrome (Figure 3(c)). Thus, based on the aforementioned findings, SAPHO syndrome was diagnosed. The clinical course of pain control using a combination of NSAIDs, bisphosphonates, and corticosteroids was good.

## 3. Discussion

Patients with SAPHO syndrome present with cutaneous lesions and osteoarthropathy, and patients with the syndrome typically present with musculoskeletal complaints, usually of the anterior chest wall, synovial articulations, and synchondroses. In young and middle-aged adults, although SAPHO syndrome is more common in the sternoclavicular region (65%–90% of patients), it can also affect the spine (33%), long bones (30%), and pelvis (13%–52%) [5]. However, the etiology of SAPHO syndrome remains unclear.

Benhamou et al. proposed that the diagnosis of SAPHO syndrome is based on the exclusion of infectious arthritis or osteomyelitis and the presence of at least one of the following four diagnostic criteria: skin manifestations of severe acne, skin manifestations of palmoplantar pustulosis, hyperostosis with or without dermatosis, and chronic recurrent multifocal osteomyelitis involving the axial or peripheral skeleton with or without dermatosis [3]. Laboratory findings such as mild, nonspecific elevation in various inflammatory indices are generally of little value. The CRP level and erythrocyte sedimentation rate are either normal or slightly elevated during exacerbations, and the white blood cell count is usually normal [6].

The current case was diagnosed as SAPHO syndrome because it met the diagnostic criteria of skin manifestations of palmoplantar pustulosis and hyperostosis and was negative for bacterial culture. In addition, based on laboratory findings, although CRP levels were initially mildly high, slight improvements were observed as clinical symptoms improved.

Radiologic findings of hyperostosis are highly characteristic of SAPHO syndrome and are characterized by chronic periosteal reaction, cortical thickening, and narrowing of the medullary canal, resulting in bone hypertrophy [7]. The external surfaces of the bone can appear expanded, and irregular joint erosions can occur because of the extension of existing osteitis [8]. X-rays of cases diagnosed with SAPHO syndrome generally reveal sclerotic lesions or mixed lytic and sclerotic lesions, and pure osteolytic lesions of SAPHO syndrome are extremely rare. Bone scintigraphy is important for diagnosing SAPHO syndrome, particularly for detecting early bone involvement [9]. SAPHO syndrome exhibits characteristic features on 18F-FDG PET/CT. Sun et al. reported that 18F-FDG PET/CT and bone scintigraphy have comparable capacity in detecting skeletal lesions [10]. In the present study, although 18F-FDG accumulation was observed on PET/CT, distinguishing between malignant tumor and SAPHO syndrome was difficult. However, differentiation between malignancy tumor and SAPHO syndrome based on 18F-FDG accumulation on PET/CT needs to be further evaluated. Bone biopsy is often performed to establish a diagnosis, especially for cases with extra-axial bone lesions that are particularly prone to radiologic misdiagnosis. However, histopathological findings of bone lesions vary, with early lesions characterized by the presence of polymorphonuclear infiltrate.

Treatment options for SAPHO syndrome include NSAIDs; antirheumatic drugs, such as colchicine, corticosteroids, and bisphosphonates; and disease-modifying agents such as methotrexate, sulfasalazine, and infliximab [11]. NSAIDs are generally considered as the first-line treatment for SAPHO syndrome, whereas disease-modifying agents are only administered when symptoms persist for at least 4 weeks despite adequate NSAIDs therapy. Bisphosphonates, which inhibit bone resorption and turnover, are frequently used as disease modifiers and anti-inflammatory agents for SAPHO syndrome [12]. Amital et al. reported that pamidronate appeared to be a very effective therapy for patients with SAPHO syndrome because it promoted remission in all affected body parts, such as the bone, joint, and skin, and ceased the bouts that characterized the disorder. Furthermore, Abourazzak et al. recommended administration of anti-TNF alpha agents, especially etanercept, in patients with SAPHO syndrome not responding to conventional treatment [13]. In patients failing anti-TNF alpha agents, IL-1 inhibitors could be an effective therapy [14]. In the current case, a combination of NSAIDs, bisphosphonates, and corticosteroids was very effective for pain control.

In conclusion, we report a rare case of SAPHO syndrome with a purely osteolytic, not osteosclerotic, lesion, leading to the misdiagnosis of a malignant tumor. This is the first report to demonstrate the radiologic change of a purely osteolytic lesion to an osteosclerotic lesion over time in SAPHO syndrome, and this knowledge of atypical radiologic findings is necessary to diagnose initial SAPHO syndrome.
